# ECRIN – CESSDA strategies for cross metadata mappings in selected areas between life sciences and social sciences and humanities

**DOI:** 10.12688/openreseurope.16284.1

**Published:** 2023-10-20

**Authors:** Christian Ohmann, Katja Moilanen, Mari Kleemola, Steve Canham, Maria Panagiotopoulou

**Affiliations:** 1 European Clinical Research Infrastructure Network, 30 Bd Saint-Jacques, Paris, 75014, France; 2 Finnish Social Science Data Archive, Tampere, 33014, Finland

**Keywords:** social sciences and humanities, clinical research, clinical trials, metadata, crosswalk, cross-domains, contextual metadata, COVID-19

## Abstract

**Background:** The recent COVID-19 pandemic dramatically underlined the multi-faceted nature of health research, requiring input from basic biological sciences, pharmaceutical technologies, clinical research), social sciences and public health and social engineering. Systems that could work across different disciplines would therefore seem to be a useful idea to explore. In this study we investigated whether metadata schemas and vocabularies used for discovering scientific studies and resources in the social sciences and humanities and in clinical research are similar enough to allow information from different source disciplines to be easily retrieved and presented together.

**Methods:** As a first step a literature search was performed, exemplarily identifying studies and resources, in which data from social sciences and the humanities have been usefully employed or integrated with that from clinical research and clinical trials. In a second step a comparison of metadata schemas and related resource catalogues in ECRIN (European Clinical Research Infrastructure Network) and CESSDA (Consortium of European Social Science Data Archives) was performed. The focus was on discovery metadata, here defined as the metadata elements used to identify and locate scientific resources.

**Results:** A close view at the metadata schemas of CESSDA and ECRIN and the basic discovery metadata as well as a crosswalk between ECRIN and CESSDA metadata schemas have shown that there is considerable resemblance between them.

**Conclusions:** The resemblance could serve as a promising starting point to implement a common search mechanism for ECRIN and CESSDA metadata. In the paper four different options for how to proceed with implementation issues are presented.

## Introduction

The recent COVID-19 (coronavirus disease of 2019) pandemic dramatically underlined the multi-faceted nature of health research, requiring input from basic biological sciences (e.g. viral characterisation and genomic sequencing), from pharmaceutical technologies (e.g. development and manufacture of mRNA vaccines), from clinical research (e.g. observational and interventional studies of candidate treatments), from social sciences (e.g. understanding vaccine hesitancy, examining political responses to the pandemic), and from public health and social engineering (e.g. promoting ‘lockdown’ behaviours and mask wearing).

The pandemic and efforts to treat it not only generated huge amounts of scientific work, results, and data, it also did so in a very wide variety of scientific disciplines. This raises the question of whether, in at least a proportion of cases, work in one discipline could have been more efficiently planned and executed, or analysed more usefully, by also considering results, insights and / or data from studies in another discipline – if, for example, recruitment to a clinical trial could have been improved by explicitly addressing concerns identified by social science surveys.

In a recent paper, the marginal role of the social sciences in the COVID-19 pandemic was stated ^1^. It was demonstrated that policymaking during the COVID-19 pandemic has been biomedicine-centric in that its evidential basis marginalised input from non-biomedical disciplines. It was argued in particular that the social sciences could contribute essential expertise and evidence to public health policy in times of biomedical emergencies and that we should thus strive for a tighter integration of the social sciences in future evidence-based policymaking. 

We are not talking about multi-disciplinary teams working on the same problem here, but rather whether knowledge and use of research outputs from one or more ‘external’ research disciplines can usefully be applied by a mono-disciplinary team. Specifically, we consider whether clinical researchers could have benefited from data and insights from the social sciences, and vice versa. As discussed above, prima facie, such extra-disciplinary ‘awareness’ ought to be potentially useful, but anecdotal evidence suggests it rarely occurs. This may be partly because researchers simply do not give consideration or are not encouraged by funders to use insights from other disciplines, or because it is difficult for people to know how and where to look for relevant resources outside of their own core discipline – i.e., the barrier to discovery is too high – or because researchers, being less familiar with the methodologies of other disciplines, are less confident in interpreting their results.

Several specialist COVID-19 portals were established, or additional pages were added to existing portals, to allow easier discovery of and access to COVID related resources, study details, results, and data. Most of these, however, were mono-disciplinary (WHO (World Health Organization) COVID page, ECRIN (European clinical Research Infrastructure Network) COVID page, CESSDA (Consortium of European Social Science Data Archives) COVID page, CLARIN (Common Language Resources and Technology Infrastructure) COVID page, others). Even when a specialist portal has been designed as multi-disciplinary and offers the ability to search across domains (e.g., the EU COVID-19 portal, the detailed metadata has remained separately organised by discipline, and often accessed via different pages. A user can therefore use this portal to discover and access research in a variety of disciplines, but they have to be comfortable with the details of the search methodology in each case. Accessing the actual datasets require several (manual) steps and data access information and procedures differ by domain and/or by dataset. In general, the current situation puts the emphasis on the individual researcher, to have both the motivation and the skills to find resources and results in disciplines outside their own, rather than having retrieval systems presenting this information automatically.

Systems that could work across different disciplines would therefore seem to be a useful idea to explore, though the initial question is whether such systems would ever be a realistic proposition. In other words, are metadata schemas and vocabularies used for discovering scientific studies and resources – whether published papers, datasets, or related material such as research protocols – similar enough across disciplines to allow information from different source disciplines to be easily retrieved and presented together, as a single search result? More accurately, are the likely costs of making metadata schemas and their underlying ontologies more congruent with each other, and thus more easily interrogated together, justifiable by the potential benefits of doing so?

It is worth noting that this question extends beyond simply having more effective and efficient searches – important though that is. Existing metadata schemas and systems were developed to meet specific and domain-centric needs, and differences between them partly reflect the different assumptions, vocabularies, research paradigms, methodologies, and available resources in those domains. If, however, metadata schemas and controlled vocabularies (CVs) were made more inter-operable, for example by mapping against each other, or by some degree of alignment (so that, for example, the same controlled vocabularies were used for some entity types), it would become easier to discover, discuss and understand scientific work across disciplines in general. This in turn could lead to an increased exchange of paradigms, metaphors, data patterns and theories that could go far beyond the simple sharing of data. In other words, a convergence of metadata could be a key enabling mechanism for a range of future work that is not easily possible or even identifiable now. While divergent metadata systems reinforce the division of knowledge, convergent schemas could help increase the chances of integrating not just data but crucially also the interpretation of that data, and the generation of new insights across disciplines.

The work was performed in the context of the BY-COVID project. BY-COVID is funded by the European Union’s Horizon Europe Research and Innovation Programme under grant agreement number 101046203. Part of the results have been presented at the European DDI (Data Documentation Initiative) User Conference (EDDI) in Paris (1 December 2022) and are published in ZENODO ^2^.

## Methods

As a first step a literature search was performed by one of the authors (CO) to exemplarily identify studies, in which data from social sciences and the humanities (SSH) have been usefully employed or integrated with that from clinical research (CR) and clinical trials (CT). This work was seen as necessary to motivate the approach of mapping metadata schemas between ECRIN and CESSDA.

In a second step a comparison of metadata schemas and related resource catalogues in ECRIN and CESSDA was performed. The focus is very much on *discovery metadata*, here defined as the metadata elements used to identify and locate scientific resources, with sufficient additional material being available for each resource to determine if it would be useful to use or at least investigate further in any particular context. A scientific resource can be a research project (or study) or a data object belonging to a research project (study), e.g., publication. *Descriptive metadata*, usually applied to datasets, includes a more detailed description of each data point, including where necessary its definition, and as such is often more domain specific. It is essential to understand a dataset once it has been retrieved or accessed, but as the focus of this report is on discovery it is not considered further here.

As basis for the comparison and mapping of the metadata schemas under investigation a generic characterisation of discovery metadata was applied. To be fully effective, discovery metadata needs to include the following metadata items ^3^:

a) A name (or names) of the resource
b) A description of the resource providing information about what the resource is, and which can therefore be used as the basis of text-based searches.
c) A persistent identifier, so that the resource can be referenced concisely and unambiguously from other systems.
d) A collection of keywords (or ‘topics’, or ‘subjects’) that indicate what the resource is about, or covers, allowing them to be used as the basis of a keyword-based search. Ideally, these keywords are constrained and / or mapped to specified controlled vocabularies (CVs), that can be used for both storing and querying the keyword data more efficiently.
e) The ‘type’ of resource, in terms of its role within the research process. Resources can be, for example, data, published papers, biological samples, result media files, study summary documents, analysis plans or web pages.
f) The ‘source entities’ described by the resource. For human related resources this means an indication of the source population, e.g. in gender, age and geographical distribution, as well as defining characteristics – e.g. a particular diagnosis, socio-economic group. Such data is useful in filtering resources within a search process.
g) The time the resource was constructed, and / or the time the underlying material (e.g. data) for the resource was assembled. Again, useful in filtering resources within a search process.
h) The methodologies used in creating the resource, in particular the nature and design of the generating study (if there is one), and the data collection methodologies used. An important part of understanding the ‘context’ of a resource, including the strengths and weakness of any resource and the conclusions generated from it.
i) The people and organisations that contributed to the resource’s creation, or that contributed to the study or activity that generated the resource.
j) Country in which the resource was collected
k) An indication of how the resource can be accessed – e.g., if it is publicly available, its location (usually as a URL) and if not publicly available how access can be requested, for example whether specific prerequisites are required, and whether such access allows downloads or is *in-situ* only. This information is especially crucial, if the resource is involving sensitive data and GDPR needs to be obeyed in detail.
l) The physical nature of the resource – its file type, size etc – and any technical details (e.g. file formats) that will need to be taken into account by resource consumers.

Most of the systems or metadata schemas include only a part of these metadata items– they represent a summary of what, ideally, should be present rather than the current reality. Resources can also be distinguished according to how they are generated and organised. For example, there is a distinction between

a) Resources originating in studies, assembled to help answer the specific set of questions that the study is designed to examine, and
b) Resources – usually data or physical samples - collected or assembled outside of any specific study, assembled as a general resource for later work, the nature of which may not be known during the resource gathering phase.

In discovery terms, study originated resources may not be easily identified outside of the context of the study – they are ‘Study X final dataset’, or ‘study Y protocol’ – and often do not have persistent identifiers or even unique titles of their own (unless they are a published paper). Discovering such *study linked* resources therefore tends to use the characteristics of the generating study to identify relevant resources – in a search context the relevant studies are found first, and the associated resources can then be examined. This blurs the distinction between the data and metadata describing studies and those describing resources. In the social sciences, particularly datasets related to studies are archived with the social science data archives/repositories. These datasets receive PIDs and all the metadata needed. It is rarer to archive datasets that are formed outside a study (e.g., register or administrative data that are usually stored and disseminated by the agencies collecting them, ^4^).

For resources collected without reference to a set of specific research questions – e.g., census data taken at regular intervals, or population level health surveys – the nature of the resource itself, usually a dataset, needs to be used in determining its relevance, e.g., its geographical and temporal distribution, and the methodology used to collect it. Such study independent resources are also much more likely to have their own unique names and descriptions, and often persistent identifiers as well. It is possible, however, to define the resource collection process itself as a ‘study’ (it depends how a ‘scientific study’ is defined), so that these resources can also be viewed as ‘study-linked’. That makes it much easier to organise the metadata when some resources are study-linked, and some are not. In that situation, as discussed above, attributes such as ‘period of collection’ can be linked to the data, the ‘study’ or both, and the distinction between study and resource metadata again becomes blurred.

There are some obvious exceptions to the description of discovery metadata above – for example review papers are resources derived from other resources rather than a study or data collecting activity. Some disciplines organise data into aggregate knowledge systems (e.g. genomic or taxonomic databases) rather than retaining study results as discrete ‘packets’ and therefore use different metadata schemas. Historical and cultural factors can also make expectations about resource, especially data, sharing different in different scientific disciplines, which in turn influences the amount and quality of metadata available. For example, in clinical research making data available to others – despite extensive pressure from editors and funders – still only occurs in a minority of cases and, even when it is possible, it is often only advertised by a brief statement stating, ‘reasonable requests will be considered by the investigator’. In social sciences, the first data archives were established in the early 1960s and were conceived as survey data archives although from the very beginning, the secondary analysis of the data was an important motive together with verification and long-term historical value ^5^. The summary as provided above does, however, apply to much of the metadata as collected and organised by ECRIN and CESSDA, and is therefore provided as a background for the analysis.

## Results

### Examples of combining data from studies in SSH and CT

The (unsystematic) literature search revealed the following examples:

- Using studies from SSH to support and optimise planning of CT (e.g., developing educational material, addressing key concerns of study participants, involving key participant groups) ^6, 7^
- Improving CT conduct and recruitment by considering data from SSH (e.g., engagement of participants by CT staff, addressing participants concerns and misconceptions, providing targeted information and feedback) ^8^
- Applying and generalising results from CTs to the real world involving SSH data (e.g., taking into consideration existing health systems, cultural norms and values, and the effects of low resource contexts) ^8^.
- Developing predictive models by combining studies from SSH and CR (e.g., combining survey data on attitudes towards vaccination with quantitative immunity data from an observational study to develop a more accurate epidemic spreading model) ^9^.
- Performing systematic / scoping reviews combining SSH and CR studies (e.g., combining social, behavioural, pharmacologic, and non-pharmacologic interventions in combat a disease or health issue) to provide more complete knowledge of diagnosis, treatment, and outcome ^10^.
- Increasing effectiveness of policy measures derived from CR by incorporating SSH data.
- Increasing knowledge about diagnosis, treatment, and outcome of COVID-19 by combining SSH and CT data (e.g., incorporating social and medical determinants into modelling of COVID-19 and other infectious diseases) ^11^.
- Increasing knowledge about associations and causal pathways between social capital and income inequality, and COVID-19 and other infectious diseases ^12^.
- Capturing psychological and behavioural benefits and pitfalls of intensive diagnostic testing to detect and prevent COVID-19 positive cases ^13^.

A major use case for mapping resources from SSH and CR lies in the provision of generalised evidence synthesis ^9^. For the exploration of the interventions in COVID-19 different research types and designs are applied, spanning both experimental and observational approaches. Studies may be prospective or retrospective, use hypothesis testing or hypothesis generating, cover a control group or not, be randomised or not, have individual citizens/patients or clusters/regions as primary target, be population-based or not, be primary research or based on secondary use of available data, use real data, or be based on simulations, etc. All these characteristics are of major importance in assessing the evidence for the efficacy and efficiency of interventions.

Currently, it is very difficult to discover similar research types and designs systematically across domains (e.g., social sciences, life sciences). Systematic reviews are slow to produce. Academics must work hard even to identify relevant clinical trials in publication databases: the studies are not clearly tagged, and CT researchers are rarely in connection with researchers doing systematic reviews. In the standard paradigm of evidence-based medicine, researchers collect evidence on a therapy from randomised controlled trials until it gets a green or red light. But in many situations, such trials are unethical, impractical, or unfeasible. Often, researchers have to pragmatically assess a range of different evidence — surveys, natural experiments, observational studies and trials — and stitch them together to give a picture of whether something is worthwhile. A prerequisite for taking research design and approach into consideration in evidence synthesis would be to make this information explicit and to link it to research projects (e.g., via tagging). The type and spectrum of examples identified, confirmed the usefulness of our approach to attempt a metadata data mapping between ECRIN MDR (metadata repository) and CESSDA DC (data catalogue). 

### The Metadata Schemas

The ECRIN metadata schema was first developed in 2016 and has been revised several times since ^14^. Almost all the resources in clinical research are study-linked, and there are often multiple resources per study. Therefore, the schema has to include substantial data about the generating studies as well as the linked resources, as described above.

In fact, the ECRIN schema consists of two inter-related schemas, one for studies and one for data objects (where ‘data object’ means any electronically available object, e.g., a file or web page). The *study schema* is based on the main data points used by ClinicalTrials.gov, the largest trial registry in the world, containing about 440,000 existing study entries (from a total of about 740,000). Those data points are themselves based around the core dataset required by the WHO and so – in broad terms – are also supported by the other 18 globally recognised trial registries. Trial registry data, particularly from Clinicaltrials.gov, represents the de facto standard data model for describing clinical research studies. The *data object schema* is based on the DataCite standard, extended to cover the needs of clinical researchers, specifically to provide additional data points covering:

- Location, ownership, and access arrangements for data objects, many of which would not be immediately or publicly available, and instead require an application process, usually to the study investigator or sponsor, for access to be granted.
- Links to the generating studies. Apart from journal articles most of the data objects generated by clinical research are closely linked to the study or studies that generated them, and are usually discovered using the study’s name or identifiers.

The metadata schema is primarily used in ECRIN and related scientific communities. There is no similar comprehensive schema for clinical research metadata from any other source, with the possible exception of the schema used internally by Vivli and a few other specialist clinical data repositories. The WHO’s ICTRP (International Clinical Trial Registry Platform) provides basic data on clinical research studies, using the WHO core dataset mentioned above. Nevertheless, the MDR remains the only portal, as far as we are aware, offering metadata on both clinical studies and clinical research outputs and other data objects.

CESSDA’s metadata schema for the CESSDA data catalogue (CDC profile), is available as an enhanced DDI profile. CESSDA also offers tools for ensuring that the metadata is CDC valid.

The CESSDA Data catalogue profile is a subset of the CESSDA Metadata Model (CMM) ^15^, which itself is mostly a subset of DDI ( https://ddialliance.org/Specification/DDI-Lifecycle/3.3/XMLSchema/FieldLevelDocumentation/ and https://ddialliance.org/Specification/DDI-Codebook/2.5/). Full DDI has more contextual metadata fields than CMM – for example DDI is able to describe the process of study development as a series of development activities, categorised using a controlled vocabulary. The CMM in turn has more contextual information than the CDC (CESSDA Data Catalogue) profile (e.g., 1.1.6 funding information, 1.1.10 study version, 1.3.4 universe, and 1.3.6 type of data source).

It could be argued that any general examination of social science metadata should start with DDI, or at least the CMM. In this case, we are focused on the subset of the DDI currently in use, as represented by the CDC profile. Therefore, only the CDC profile is used for most of the comparison with the ECRIN clinical research metadata schema.

***The Catalogues.*** ECRIN provides MDR, which is a database and associated web portal that brings together metadata data at a global level about a) clinical research – the studies – and b) the resources linked to them – the data objects and makes that metadata searchable in various ways ( https://crmdr.org/). The MDR and its metadata schema are both described further at ( https://wiki.crmdr.org/index.php?title=Project_Overview). Currently, the MDR and portal are being rewritten and the material will be updated. The rewrite is also intended to add a public API to the MDR. The system currently has data on about 740,000 studies and 1.1. million objects.

CESSDA maintains a multilingual Data Catalogue CDC ( https://datacatalogue.cessda.eu/), which contains metadata relating to more than 40,000 datasets held by CESSDA’s Service Providers (SPs). The Service Providers are national data archives and repositories from CESSDA member countries. The CDC is designed as a ‘one-stop shop’ for searching and finding European social science data. 

In both systems resources are linked to studies. In the MDR all studies are registered clinical research investigations – about 80% are interventional (i.e., clinical trials), and almost all the other are observational studies of various kinds. A very small proportion (several hundred in total) are listed as ‘expanded access’ (case studies of compassionate use of novel products) or ‘registries’, (collections of routine health data). In CESSDA, most resources are also linked to studies. Even when this is not strictly the case, the resource is treated as if it were linked to a study – for example entries labelled as censuses are linked to a ‘study’ that is the name and year of the census. This allows the same study-resource metadata schema to be applied to all entries in the system.

There are also similarities in the way in which the data is presented. In both systems a search using words or categories provides search engine result page (SERP) listing all suitable studies, paginated to 30 by default in the CDC, 10 in the MDR. In both cases users can navigate from any listed study in SERP to a record/item level details page that provides a more complete description of the study and the linked data object(s). In the CDC, the user can navigate from the details page or the SERP to the service provider page, i.e., external to the CDC, that provides further details about the dataset and how to access the dataset itself. Accessing the dataset often requires the user to log into the information system of the CESSDA Service Provider, implying the need to set up an account for that specific system. In the MDR, the user can navigate, from the details page or the SERP, directly to publicly available resources. If a resource is under a controlled access, the user should normally be able to navigate to a page giving the information about how the resource can be accessed. A key public resource in the MDR is the study registry page (sometimes two or more registry pages are present) that includes the public details of the study. In some ways, these pages are analogous to the SP page that can be accessed via the CESSDA Data Catalogue, although the detailed (meta)data available on each is very different.

A key difference between the two systems is that in the MDR a study can be linked to several resources – a study registry page as a minimum, often a results summary, papers published about the study, a study protocol etc. Of the 1.1 million objects in the system, about 750,000 are study registry pages, leaving about 350,000 that are some other types of resource – over 200,000 are associated journal papers. The number of linked data sets, however, is very small, about 1200, because data sharing in clinical research is a recent phenomenon and many researchers are still uncertain about exactly how and when it should be done. A recent survey by ECRIN of COVID-19 studies found that although about 15% of researchers in those studies made some form of commitment to data sharing, only a tiny proportion of that number had actually provided any data ^16^.

In contrast all entries in the CDC are linked to a single dataset, though that might consist of several files, and in some cases associated with information about publications e.g., journal papers where the dataset has been used. In some cases, the ‘study’ is to be defined by the dataset, to maintain that one-to-one link. For example, there are seven studies named ‘Effects of the Trades Union Studies Project, 1977-78’ followed by a specific sub-study name, and each has an associated dataset of the same name. In MDR terms, this would be one study with 7 datasets, each corresponding to sub-studies, in CDC terms it is 7 studies each with a single dataset.

Another difference between MDR and CDC is that CDC has multilingual metadata. The metadata is in the languages of the Service Provider, though in many cases it is provided in both English and in the local languages. Currently, about 75% of study descriptions are available in English in CDC while MDR contains metadata only in English. 

The difference between the two systems might be summarised by saying that the MDR is *study-led*, and identifies data objects linked to those studies, whereas the CDC is *resource-led*, and identifies (or sometimes even defines) a study as the activity that created the resource. This is not surprising given that the MDR starts with study registry data, whilst the CDC starts with data archives and their contents. 

### Retrieval of resources

Many of these advantages mentioned in the introduction were particularly identified in the context of COVID-19 but most of them would apply to any condition. For this reason, there does seem to be genuine added value in being aware of, and using, CR and SSH data together. To investigate this question further, a series of quick searches were carried out – in the CDC using health-related terms, and in the MDR using social science terms (taken from the CESSDA topic list).

Table 1 shows the numbers of studies retrieved from the CDC using the selected health-related terms. As one would expect the more specific the term the fewer the studies found. The studies found would obviously need to be investigated further, but in all cases the numbers represent a non-trivial set of potential resources.

**Table 1.** Results of searching (in English only) for health-related terms in the CDC. (accessed 13 February 2023).

Term	CESSDA (from 26,362 entries in English)
health	8271
health service	3710
covid	781
heart	355
heart disease	104
nutrition	677
drug abuse	886
schizophrenia	21

Table 2 shows similar results, showing the number of studies found with ‘social science terms’ in their title or listed keywords in the MDR. The results here are perhaps less impressive given the much greater size of the MDR, and as above any preliminary results would need investigating further. Both searches were, however, very quick exercises using single terms rather than a cluster of synonyms, and a more rigorous search would almost certainly yield increased numbers, in both cases.

**Table 2.** Results of searching for social science related terms in the MDR.

Term	MDR (from about 740,000 studies)
attitude	1603
minorities	531
trust	244
crime	85
quarantine	76
occupational health	38
social change	35

If we look specifically at COVID-19 in the MDR, we get more than 10000 studies with a search of “COVID-19” in the title. If this is restricted by filtering to “interventional trials”, 7185 resources remain (22.02.2023). In the CESSDA data catalogue a search for “COVID-19” finds 750 studies from a total of 36758 studies in English. If we look further down to specific interventions, we can identify with a search for the term “lockdown” 95 resources in CESSDA and 76 studies in the MDR. This example shows that even for a specific intervention, quite a few biomedical and social studies can be identified.

### Detailed comparison of Metadata between CESSDA (CDC), ECRIN (MDR) and fundamental discovery metadata items

Given the clear potential for using metadata from both systems, the next question is how easy it would be to do using an integrated search mechanism of some kind including the discovery metadata items defined in methods section. As already mentioned before, CESSDA Data Catalogue contains study-level metadata at present. Therefore, it is possible only to compare metadata items in the context of study but not in the context of a data object. One of us (SC) performed a ‘cross walk’ between the MDR metadata schema and the CDC metadata schema, later annotated and clarified by a colleague from CESSDA (KM). The crosswalk is openly available in ZENODO ( https://zenodo.org/record/8129621). The walk was performed in both directions, i.e. if and how CDC metadata could be integrated into MDR, or MDR metadata integrated into the CDC. Then we checked if MDR and CDC metadata has all the discovery metadata items listed in methods. 

The findings of the ECRIN (MDR) and CESSDA (CDC) metadata mapping to the discovery metadata specified in methods are shown in Table 3.

**Table 3.** ECRIN – CESSDA discovery metadata.

Discovery Metadata Item	MDR (ECRIN)	CDC (CESSDA)
A name (or names) of the resource	Yes (Usually public and scientific titles, often also acronyms)	Yes (Study title)
A description of the resource	Yes (Study description, outcomes, end-points)	Yes (Abstract)
A persistent identifier	Partly (Identifiers, persistent in practice but not formally guaranteed / structured as PIDs)	Partly, some of the records include PID some only identifier (Study number / PID)
A collection of keywords (or ‘topics’, or ‘subjects’)	Yes, but vocabularies vary with source and keyword type	Yes (Keywords, Topics)
The ‘type’ of resource	Yes	Not explicitly
The ‘source entities’ described by the resource	Yes, (Participants specified by gender, age, inclusion- and exclusion criteria)	Yes (Analysis unit)
The time the resource was constructed	Partly (Start of the study is included - end dates may be be present but often approximate)	Yes (Data collection period)
The methodologies used in creating the resource	Yes (e.g., study phase, allocation type, intervention model, masking; but not always complete)	Yes (Time dimension, Sampling procedure, Data Collection mode)
The people and organisations that contributed to the resource’s creation	Yes (Trial sponsor and study leads / contacts usually identified, also additional funders)	Yes (Creator)
Country in which the resource was collected	Yes (Country)	Yes (Country)
An indication of how the resource can be accessed	Yes, (Through data sharing statement but access for datasets not always explained	Yes (Terms of data access and link to the resource)
The physical nature of the resource	No (Only in a small minority of cases)	No

It is considered how each of the main elements of discovery metadata are treated in these two systems and therefore the potential interoperability between them. The main points to emerge are:

- The first three discovery metadata items a) study name(s), b) description and c) identifiers appear in these two systems broadly similar, and it would be straightforward to (for example) use this metadata from one system inside the other or access it in both systems with a common interface. A major difference is that the datasets referenced by CESSDA usually have a PID (persistent identifier), often a DOI (digital object identifier), whereas the objects in the MDR, except journal papers, usually do not. In addition, objects in the MDR (again the major exception is journal papers) often do not have a pre-specified name, and so one must be constructed for display and listing purposes, whereas the objects in the CDC all have a title, usually the same as or related to the study name.
- The discovery metadata item d) a collection of keywords occurs both in MDR and CDC. Nevertheless, the most significant difference between these two systems lies in the *controlled vocabularies* (CVs) used to generate keywords or topics. While the ways CVs are used are very similar in both systems, the CVs themselves are not. Within the MDR most topics are taken from MeSH (Medical Subject Headings), reflecting their use in two important US data sources – Clinicaltrials.gov and PubMed. If MeSH cannot be used the topics are usually as generated by the authors / study leads, though a few use MedDRA or ICD for ‘condition under study’. In CDC there are both ‘Topics’, using the relatively high level CESSDA topic classification, and keywords using the ELSST (European Language Social Science Thesaurus), SPs’ national thesauri or keywords given by authors.
  The ELSST includes about 3,300 concepts covering social sciences with the most of them available in each of 16 languages. In contrast, MeSH has about 30,000 heading terms covering all aspects of medicine, and another 300,000+ supplementary terms (although a lot of these are simply the names of chemicals and medicines) but it is almost entirely in English. MedDRA, for comparison, covering medical diagnosis and symptomology, has about 25,000 concepts (‘preferred terms’) and about 85,000 synonyms, and claims to be translated into 15 languages. Finally, the WHO’s International Classification of Diseases (ICD), again designed to be used across languages, has a full system of about 35,000 terms, but by using only the 4 character ‘stem’ codes, this can be reduced to about 4,800, and that can be reduced further, to a few hundred terms, by considering only the ‘blocks’ of related codes.
- The type of the resource (discovery metadata e) is not part of the metadata in CDC but it is explicitly stated that CDC is Data Catalogue. In MDR, the type of resource is part of the metadata and included under “type” of data object attributes.
- The discovery metadata item f), the source entities, which is the description of the population appears both in MDR and CDC. Still, it is handled in different ways in these two systems, reflecting the different levels of specificity that are usually required.
- Study location, country in which the resource was collected (j), is present in both systems, but it is more explicit in the CDC
- The time the resource was constructed (g), the start and end times of data collection are captured more clearly in the CDC. In the MDR they often are estimates in the prospective study registration records.
- There are several metadata items related to discovery metadata h), the methodology. One of them, occurring both in CDC and MDR, is study design type: whether the resource is produced in an observational or interventional manner. In the CDC, almost all the studies are observational (mostly surveys) while in MDR most of the studies are interventional. This is reflected in both metadata schemas.
- With respect to i) study contributors (people and organisations), these two systems are mainly similar but have differences in detail. At the present, there is only Creator (author, principal investigator) in CDC. In MDR, for example, the sponsor, i.e., the organisation that has legal responsibility for a clinical trial, exists. In social sciences, the controller has legal responsibility on data protection but usually this information is not recorded in the public metadata
- The crucial discovery metadata item for the users, an indication how the resource can be accessed (k) is available in both of the systems, MDR and CDC. They both have a metadata field for describing data access (CDC “Terms of data access” and MDR “Data sharing statement” and “location and access details”).
- The physical nature of the resource (l) is missing both from the CDC and MDR.

### Mapping of clinical research “study types” between CESSDA and ECRIN

In the paper of Sim *et al.* ^17^, a human study design ontology for clinical research is presented. Through iterative consultation with statisticians and epidemiologists, the typology of study designs has been based on discriminating factors that define mutually exclusive and exhaustive study types In Table 4, the classes belong to study design in OCRE are presented ( https://bioportal.bioontology.org/ontologies/OCRE/?p=classes&conceptid=root):

**Table 4.** Classes belonging to “study design” in OCRE. (*from: https://bioportal.bioontology.org/ontologies/OCRE/?p=classes&conceptid=root).

Ontology of Clinical Research (OCRE *)
• Case-only study design • Qualitative study design ◦ Case series study design • Quantitative study design ◦ Interventional study design ▪ Crossover study design ▪ N-of-1 crossover study design ▪ Parallel group study design ▪ Single group study design ◦ Observational study design ▪ Case-control study design ▪ Case-crossover study design ▪ Cohort study design ▪ Natural history study design ▪ Cross-sectional study design

### Considerations regarding documentation of “study type” in ECRIN and CESSDA

The considerations should help to clarify whether specific clinical research study design can be documented in CESSDA and as well in the MDR. This could be of value for generalised evidence-synthesis cross social sciences & humanities and clinical research.

***Interventional studies.*** In the MDR, there is a primary distinction between interventional trial and observational study. This is reflected in the MDR filter variable “Type” (interventional, observational). In CESSDA, most of the studies are observational without intervention. Interventional studies are characterised by using DDI Alliance Controlled Vocabulary for Mode Of Collection and the term “Experiment “ or even more specific “Field/Intervention experiment”, “Laboratory experiment” and “Web-based experiment”. In the term definition of “ Field/Intervention experiment“, interventions/clinical studies are explicitly mentioned.

In clinical research participants are selected according to defined inclusion and exclusion criteria, which are defined in the study protocol. This is usually a non-randomised procedure with no random sampling from a larger population, so it is not explicitly documented. Instead, for clinical research, the type of allocation of research participants to the interventions is of major importance. This can be documented in the MDR, for example, by “allocation type = randomised”. In CESSDA, the selection of research participants would be documented from the beginning. There should be at least two different terms for the sampling procedure (using DDI Sampling Procedure CV) and also some free-text explanations. First there would be a term telling which kind of Non-probability (non-randomised) sampling is done e.g. "Non-probability: Purposive" and additional text e.g. "At the first selection phase of research participants, the research participants were chosen based on their suitable medical history." Secondly there would be a term telling that this group of participants selected in the first phase of sampling, is in the second sampling phase divided in two (or more) different groups using Probability (randomised) methods e.g. "Probability: Simple random" and adding again explanation text e.g. "In the second selection phase, the selected group of research participants were divided into control group and intervention group with simple random sampling."

Another important aspect may be the observation unit of the study. In clinical research, most of the trials are with individuals but some trials work with clusters, e.g., cluster-randomised trials. This is not used in the MDR but in CESSDA, the analysis unit can be e.g. “Individual”, “Event/Process/Activity”, “Organization/Institution”, “Group” or “Family” as the DDI Alliance Controlled Vocabulary for Analysis Unit is used.

***Observational studies.*** In the MDR, observational studies can be identified by “type = observational”. In CESSDA, this can be documented by using the DDI Alliance Controlled Vocabulary for Mode of Collection and the term “Observation” or with more detailed terms e.g. “Field observation”, “Participant laboratory observation”. Here specifically, development of condition or disease is mentioned. The narrower term “Laboratory observation” is relevant when comparing to clinical research.

In clinical research, there are several subtypes of observational studies. For clinical trials, of primary interest are **cohort studies**. In MDR, this is covered by “observational model = cohort”. In CESSDA this can be documented via using DDI Alliance Controlled Vocabulary for Time method and the term “Longitudinal: Cohort/Event-based”. Other types for clinical trials are **case-control**, **cross-section studies and case series**. In MDR, case-series are covered by “observational model = cases only”. There is no counterpart for case-series in the vocabularies used by CESSDA. The term with closest match is “Non-probability: Purposive” from DDI Sampling Procedure vocabulary describing that the research participants were selected for the information they can provide on the research topic. To be able to identify case-series studies, there is a need to have some additional text explaining the study design/sampling. MDR has included “observational model = cross-sectional”, which is described in CESSDA by using DDI Alliance Controlled Vocabulary for Time method and the term “Cross-section”. With respect to case-control study, there is “observational model = case-control” in the MDR. Case-control cannot be directly described with vocabularies used by CESSDA. The best option would be the same as with case-series, to use the term “Non-probability: Purposive” from DDI Sampling Procedure CV and to add again additional text explaining the study design/sampling.

Trials with simulation can be documented in CESSDA by using DDI Alliance Controlled Vocabulary for Mode Of Collection and the term “Simulation“ but not in the MDR.

The possibility to document specific clinical research “study types” in CESSDA and ECRIN MDR is summarised in Table 5:

**Table 5.** Documentation of specific clinical research “study types” in CESSDA and ECRIN.

Study type	CESSDA	ECRIN MDR
Interventional	+	+
- Randomised	+	+
Observational	+	+
- Cohort study	+	+
- Cross-section	+	+
- Case-series	-	+
- Case-control	-	+
- Randomised sample	+	-
Simulation studies	+	-

## Discussion

Given the differences and similarities between the metadata schemas and systems of ECRIN and CESSDA, and the underlying studies and data objects, the question is what might be desirable and possible in discovery metadata integration, and what further work might be needed for clarifying these questions. Here in the discussion, we give tentative suggestions which will require further discussion. These suggestions are made largely with reference to the existing CDC profile, but the crosswalks demonstrated that the bulk of the potential interoperability between the clinical research and social science metadata exists within this profile. Using the CMM instead of the CDC profile would therefore add comparatively little to the current discussion.

The following suggestions are made:

- With respect to names (a), identifiers (c) and descriptions (b) the metadata schemas of CESSDA and ECRIN appear broadly similar, and it should be straightforward to map these metadata items from one schema to another. Interpretations and definitions of each of the metadata items would need to be clarified and checked but no substantial problems are foreseen.
- The explicit type of the resource (e) is implicit in CDC and explicit in MDR. Mapping of the type of the resource should be effortless and machine-actionable.
- The source entities (f) are described both in MDR and CDC, but the level of detail is different. This might cause troubles, if we wished to harmonise this metadata. MDR has more detailed fields for describing the source entities but they are rarely filled in. CDC has only one field describing source entities: the unit of analysis (e.g. individual, media unit).
- As part of the methodology (h), the type of the study is essential especially for the clinical research. At a high level, it ought to be possible to categorise study *types* into a single comprehensive system that is able to indicate
  a) whether or not a specific research question or hypothesis was being examined, as opposed to data collection on a routine, periodic basis, (be that in the general population or in a specified subgroup and / or setting such as a hospital). This actually can be a bit challenging because there is not a proper field for this kind of information in CDC profile (or DDI). Most of the studies/datasets available from CESSDA are collected because of the specific research question and only a very limited amount has another origin, e.g., census.
  b) whether the methodology used was interventional, observational or something else. In ECRIN this information is available, in CESSDA “mode of collection” could be a starting point for doing this classification.
  The difficulty here is that this would involve additional work in classifying the CESSDA studies, the distinction already being made within the MDR, and thus is unlikely unless the effort was thought to be worthwhile.
  At a lower level of ‘study type’, ECRIN may benefit from exploring the usage of the CESSDA categorisations for observational studies, referring to the mode of collection vocabulary (though the methodological details are often not available in the source data). CESSDA could utilize some of the interventional study classifications in the MDR schema (which are taken from ClinicalTrials.gov), but again it would depend on the work involved and whether the details were present in the source data. It should be taken into consideration that interventional studies in social sciences are very rare.
- In MDR, other metadata items concerning methodology are dependent on the type of the study, whether it is observational or interventional. In CDC, both these study types have the same metadata items. For observational studies MDR uses vocabularies “Observational study model” (metadata item “Observational model”) and “Time perspective”. In CDC, the same kind of information can be found from the “Data Collection Mode”, “Time Dimension”, and “Analysis Unit”. There is also “Sampling procedure” in CDC, but it does not have a counterpart in MDR.
  For interventional studies MDR has “Phase”, “Primary purpose”, “Allocation”, “Intervention model” and “Masking”. In CDC, the “Sampling procedure” includes the same kind of information as “Allocation” in MDR, but other MDR metadata items are out of scope of the CDC. In CDC, also “Data collection mode”, “Time dimension” and “Analysis unit” are used for interventional studies too.
  The difference of these two disciplines can be noticed very clearly in the methodology. Some of the information can be mapped, but not all. The ideas of how to do the mapping is included in the appendix.
- Metadata items representing person and organisation (i) contributors could probably be mapped without too much difficulty. Persistent identifiers (PIDs) e.g., ROR (research organization registry) for the organisations and ORCID (Open Researcher and Contributor ID) for persons, could help to make the metadata more consistent and searchable. Persistent identifiers are useful but the proportion of source data containing them continues to be relatively low. This is at least partly due to the costs and effort needed to update a considerable amount of legacy (meta)data.
- Metadata items for study dates (g) could also be mapped but not the date when the resource was constructed because it is often missing from MDR. Unfortunately, the date when the data became available is the only one present in both schemas. Other dates than the year of publication / availability, would be difficult to map.
- Study location could probably be mapped at the level of the country (j) but not more detailed than that. A consistent set of identifiers for countries (e.g., from Geonames) could help with metadata consistency.
- Both MDR and CDC have keywords (d). To achieve a more consistent use of keywords, it might be possible to explore an approach where different CVs (controlled vocabularies) were used for different types of keywords. Thus,
  - For terms describing diagnosis or ‘relevant medical condition’, it might be possible to use ICD (International Classification of Diseases) 11 as the master CV and map other terms to that. This is currently being explored in the context of the MDR, where the plan is to provide an ICD browser as part of the search mechanism.
  - For terms covered by the ELSST (European Language Social Science Thesaurus), it might be considered as master CV, with other terms mapped to that. Again, ideally, a browser to navigate the thesaurus should be available as part of a search mechanism.
  Similar approaches could be used for other ‘domains’ of terms. Quite common approach is to do the mapping between the different CVs used but it is both difficult and expensive. Rather than trying to use a single CV that suited everyone in every case the longer-term solution is to push the source systems into using a common set of CVs. In the past, that has proven to be very difficult, and certainly ECRIN has no power to do that with respect to the source trial registries it uses. It is suggested that exploring the feasibility and practicality of ‘CV convergence’ would be a useful next step in examining how metadata can be made more inter-operable, even if the conclusions are that the resources required would need substantial amount of additional funding.
- As stated previously, the physical nature of the resource (l), is missing from the both (MDR and CDC).

If we wished to integrate the search mechanism of both systems, and assumed that the metadata schemas themselves could be made sufficiently interoperable, there are at least four implementation possibilities:

a) Include CDC search result metadata within the MDR.
b) Include MDR search result metadata within the CDC.
c) Provide a new, single search interface ‘over the top’ of both systems, with the results integrated to some extent, but with each returned study linking back to details in the source system.
d) Include the metadata of both systems into an overarching system (e.g., COVID-19 Data Portal, OpenAIRE)

First three approaches would require an effective search API (Application Programming Interface) to be available for both the MDR and the CDC – there is no suggestion that the metadata should or could be integrated at the level of the source data. Instead, it would be the search results that would be integrated. The difference between these three systems is therefore mainly in the location and nature of the search mechanism and the location of the listed results.

The CDC is part of the EOSC (European Open Science Cloud) marketplace and has recently developed a search API ( https://api.tech.cessda.eu/ and https://api.tech.cessda.eu/#/DataSets/findRecordsByQuery). This API supports better interaction with external collaborators and makes it possible to search over all the records in the catalogue. CESSDA also has an OAI-PMH (Open Archives Initiative Protocol for Metadata Harvesting) endpoint that exposes the metadata in DublinCore, DataCite and DDI 2.5 Codebook formats. Currently, around half of the resources is covered by OAI-PMH.

The CDC metadata generally appears more consistent than the MDR metadata. CDC is currently focused on datasets, while MDR has studies with only other types of linked objects. Therefore, including MDR metadata into CDC search engine result page could confuse users. Conversely, integrating the CDC search result metadata within a MDR SERP (search engine results page) might be easier. MDR already contains a wide range of different data objects attached to studies. Links in such a case could lead back to the CDC details page, as now in the CDC, or possibly back to the SP page, so that access to the data was more immediate. Accessing the dataset would, in many cases, require logging into the CESSDA service provider’s information system. 

The option of having a different search portal altogether, in a sense overlaying both systems, is probably the most attractive. It would allow search results to be returned either as a single integrated list or as two lists within the same page. The important thing is that both lists would be automatically visible to the user. The major difficulty is that it would require some developer input, as well as separate and additional hosting arrangements. The hosting should not be too difficult, but developer input is often difficult to resource within academic research infrastructures, so setting up such a portal might be more difficult than simply extending one of the existing systems.

The fourth option is to link resources from different domains to overarching system like COVID-19 Data Portal and the OpenAIRE Research Graph. The COVID-19 Data Portal facilitates data sharing and analysis by bringing together and continuously updating information about relevant COVID-19 datasets and tools. The Portal allows users to search across all different data types presented on the Portal. Data sources include from molecular biology and clinical data to social sciences, empowering researchers’ discoveries of relevant data objects from potentially unfamiliar resources. OpenAIRE harvests metadata not only about publication but about research products in general, covering datasets, software, and other research products. In the OpenAIRE Research graph, research products are related to projects, data sources, funders, organisations, beneficiaries, etc. It would be beneficial to add metadata from domain-specific repositories, such as the metadata repository (MDR) of ECRIN. CESSDA DC is already part of the OpenAIRE Research graph.

With respect to this fourth option, some work has already been done. CESSDA Data Catalogue is one of the COVID-19 Data Portal’s as well as OpenAIRE’s metadata sources. The MDR currently does not provide public APIs or endpoints, but this is under development and will come soon. It is planned to provide a RESTful API and a GraphQL API. Development of an API will allow interoperability with OpenAIRE. There is also an ongoing mapping of the MDR with the OpenAIRE RG (Research Graph). Therefore, the progress of the integration with OpenAIRE is expected soon. However, not all metadata items from CESSDA DC and ECRIN MDR have a direct counterpart in OpenAIRE and have to be handled as descriptive text. Unfortunately, this will result in some loss of structured information for discovery.

## Conclusions

A close view at the metadata schemas of CESSDA and ECRIN and the basic discovery metadata items presented in methods, as well as a crosswalk between ECRIN and CESSDA metadata schemas have shown that there is considerable resemblance between them. The resemblance serves as a promising starting point to implement a common search mechanism for ECRIN and CESSDA metadata. In this paper, we present four different options for how to proceed with the implementation issue. Either way, further discussions and the provision of use cases demonstrating the benefit of the approach to be selected will be needed. Regardless of the selected implementation, there could be support for discoverability of research types and designs. This approach has a huge potential to improve predictive models, to increase knowledge and to provide better evidence synthesis with data coming from different domains, in this case from life sciences and social sciences & humanities.

## Data Availability

The crosswalk underlying the analysis between CESSDA Data Catalogue (CDC) DDI2.5 Metadata Profile ( https://cmv.cessda.eu/profiles/cdc/ddi-2.5/1.0.4/profile.html) and ECRIN Metadata Schema for Clinical Research Data Objects Version 6.0 (August 2021) ( https://zenodo.org/record/5554961) has been registered with the DOI 10.5281/zenodo.8129620 and with the licence Creative Commons Attribution 4.0 International in ZENODO ( https://zenodo.org/record/8129621).
