# Supplementary material for: ECRIN – CESSDA strategies for cross metadata mappings in selected areas between life sciences and social sciences and humanities
Source: Open Res Eur. 2023 Dec 11;3:180. Originally published 2023 Oct 20. [Version 2] doi: 10.12688/openreseurope.16284.2 (PMC10643878; doi:10.12688/openreseurope.16284.2)
Supplement: Peer review history

# Reviewer report: Reviewer response for version 1

**DOI:** 10.21956/openreseurope.17579.r35761

**Published:** 2023-11-13

**Authors:** Ricarda Braukmann

**Affiliations:** 1 DANS-KNAW, Nijmegen, The Netherlands

Summary of the article

 The paper “ECRIN – CESSDA strategies for cross metadata mappings in selected areas between life sciences and social sciences and humanities” by Ohmann and colleagues describes the comparison of metadata standards and vocabularies used in the Metadata Repository of ECRIN (MDR) and the Data Catalogue of CESSDA (CDC). With the background of the COVID-19 pandemic, the authors pose the question whether researchers in the clinical domain could have benefited from insights and data from the social sciences, and vice versa. A role is seen for retrieval systems that operate across different disciplines and the authors want to assess the resemblance between the metadata and vocabularies used for resource discovery in two exemplary domain-specific systems.

 The authors performed a literature search and they compared the metadata from the MDR and CDC. They assessed the search system, as well as creating a crosswalk between the two metadata schema’s and the authors systematically compare how a selection of 12 discovery metadata elements are implemented in each system.

 The authors observe that with respect to names, identifiers, and descriptions the metadata schemas of CESSDA and ECRIN appear broadly similar, whereas some information related to the methodologies in the disciplines is harder to map to each other.

 In the discussion section, proposals are made for how existing elements and Controlled Vocabularies (CVs) could be used to align the metadata to improve the interoperability. The authors finish their assessment by presenting four possibilities how an integrated search mechanism could be realized (i.e. including metadata in each other’s system, creating a new system or including both in an existing overarching system) and addressing the possibilities and challenges of each option.

 Overall the authors conclude that there is a resemblance in the discovery metadata used in the ECRIN MDR and the CDC which is promising when assessing the possibilities to have a common search mechanism for clinical and social sciences data, yet the four options for implementation which are presented would need to be further assessed.

General impression

 The paper assesses the resemblance of metadata used in two domains which could benefit from more integration. I find this paper therefore a very useful exercise and a good initiative to assess how metadata from different existing domain-specific systems could be combined. The selection of clinical data and social sciences data seems very relevant when referring to the COVID-pandemic in the introduction, although this exercise could be replicated with other example catalogues from different domains as well.

 The authors assessed the used metadata profiles in a lot of detail and created a crosswalk as well as making concrete suggestions on how existing fields and CVs could be used by the other system to enhance the interoperability.

 I have some recommendations and requests for clarification which I will briefly introduce here and outline below in more detail.

 Firstly, one aspect that I was missing in the paper that I believe the paper would benefit from is including more information about the **user perspective** and considering what end user is envisaged in the common search system for which the metadata comparison is made. It would be great if the authors would elaborate on this end user perspective when comparing the two systems as well as when evaluating the four options that are presented for implementing a common search system in the discussion.

 Next to elaborating on the user perspective, I have some concerns about the **comparability of the two systems** and how the search results that are presented can be interpreted. I would like to propose some adjustments that could provide clarification.

 I also recommend to present the **literature study** in a different way.

 I have some questions regarding the **assessment of the discovery metadata elements**, in particular the PIDs, and the Data Access Terms.

 In the discussion, I would recommend a more extensive **elaboration on the fourth option** that is presented.

 Lastly, I a few **minor comments **and requests for clarification.

 One technical thing I want to note is that I was not able to view the **Appendix** that is referred to in the paper. It was unclear to me where I can download this document.

Detailed Recommendations

*Methods - Literature review*

 The literature search is presented in the abstract and introduction as the first core element of the methodological part of the study. The literature study is, however, not described in much detail in terms of the methodology that was used to execute the study and the Results section lists examples of studies that were found rather than presenting a comprehensive list of results. With how the literature search is presented in the abstract and introduction, I would have expected more information about the search engine and search terms used, the selection criteria, the number of results, and the selection for further assessment. It appears that the literature study was conducted mainly to strengthen the existing assumption that it would be useful to compare the metadata from clinical and social science portals. The authors themselves state: “This work was seen as necessary to motivate the approach of mapping metadata schemas between ECRIN and CESSDA.”

 In my view, the focus of the paper would be improved if the literature study was included as part of the introduction rather than being described in the methods and results. It seems that this was in fact part of the background research that was done to justify the main goal of the paper - namely assessing the resemblance between the metadata used in the two domain portals.

*Methods - Comparability of the Catalogues - end user perspective*

 While I do understand the reasoning behind the selection of the ECRIN MDR and the CDC for comparison, I do wonder a bit about their comparability. MDR is a registry of different resources associated with a given study, including data but also other aspects, whereas the CDC is a data catalogue.

 I wonder if both systems are used for the same purpose by an end user. In the CDC an end user can find datasets - often datasets that are available for reuse - but no other information is available, e.g. on social science publications or preregistered studies. The MDR contains more studies to browse through, but - as far as I can evaluate - many of the records to not contain a link to the underlying data. This distinction could be stressed more in the paper and should be considered when discussing the merits of combining information from the two systems. Considering different end users that such a combined system would address would be useful. I can imagine for instance that a policy officer is interested in published results whereas a researcher might be interested in the underlying data. Discussing who a combined system would be targeted towards seems a useful contextualization.

*Results - Comparability of the Catalogues*

 Due to the difference in the catalogues (see above), I also find it difficult to evaluate the comparison that is presented in the results in terms of the search results (i.e. presented in Table 1 and Table 2). A resource discovered in the CDC will always be a dataset, while a resource in the MDR will be study information that may or may not have a link to a dataset. I think it would be useful to present the results including the resource type for the MDR. I would be interested to see how many results contain links to datasets as this would make it better comparable to the CDC results presented.

*Results - Assessment of the Discovery Metadata*

*PIDs*

 I wonder about the evaluation of Persistent Identifiers in the MDR. An important aspect for the FAIRness of the metadata would be the inclusion of an identifier that is not only persistent and unique, but is also actionable so that one can connect with the original resource. It would be great if this aspect of PIDs could be included in the evaluation.

*Data Access terms*

 I find this aspect of the discovery metadata difficult to compare between the catalogues as the records in the CDC refer to datasets and include licences and data access statements, whereas the records in the MDR refer to studies where – in my understanding – not always a link to a dataset is included. I also noticed when looking at some records in the MDR that although a data sharing statement is available it is often not filled out (“NaN”). It would be great if the authors could elaborate on this aspect a bit more and also provide insights in whether certain fields in the Discovery Metadata are obligatory and typically provided in the two systems.

 For evaluating the usability of the metadata and for an assessment by the end user, I suppose this would be an important aspect.

 In addition to the access terms, I am missing an evaluation about the licence and whether a resource is openly available. This seems to me to be an important aspect of the access information for a particular resource.

*Discussion - Generic versus topic-specific catalogue*

 I wonder if the fourth option that is presented should not make a distinction between an overarching system that is specific to a certain subdomain or topic, like the COVID-19 portal compared to an overarching system that is domain-agnostic like the OpenAIRE graph.

 In the discussion of these different options, I also miss the user perspective. The options are outlined in terms of technical feasibility, but I would like to hear the authors perspective about the envisoned end user and in what way that perspective influences the choice of system in which the combined metadata would be presented?

 I also wonder if these four options are mutually exclusive or if different options would be considered depending on the end user in mind.

 With respect to the fourth option of integrating both into an existing system, I was wondering whether CDC and ECRIN metadata are already mapped to a common standard. It would be great if the authors could include information about existing crosswalks between the CDC, ECRIN metadata and other standards. I would particularly be interested in existing mappings to OpenAIRE and schema.org or DCAT. Including these in the supplementary materials or providing a persistent link to an existing mapping would be a useful addition to the paper.

 As OpenAIRE is explicitly mentioned I would also be interested to know how the assessment of the two systems relates to what is available in OpenAIRE with respect to discovery metadata elements that were assessed. The authors mention that not all items can be mapped but it would be nice if more specific information was included, for instance in the mapping that is mentioned to be in the Appendix. I do have to note that I was not able to find the Appendix in the pdf of the paper or online so I was not able to evaluate what information is already included.

Other comments

 - The article says to focus on the life sciences and social sciences *and humanities,* yet the data that is assessed is from the clinical research field and from the social sciences. Examples provided in the introduction seem to be mainly social sciences research (psychological behavioral data) and I miss the humanities perspective. Therefore I would recommend to rephrase the title and the reference to Social Sciences and Humanities to only include Social Sciences. I understand that SSH is often used as a term to describe the wider domain, but in my opinion referring to social sciences covers better what was assessed in this paper. Similarly, one might want to consider rephrasing life sciences to clinical research, yet as this is not my area of expertise I am not sure how this would be perceived by life science researchers.

 - It would be useful to include a list of abbreviations at the end of the document.

 - In table 3, it would be helpful if the letters the authors use for the discovery metadata in the Methods section is also added in the table as the letters are used in the text and this would be helpful for referencing to Table 3.

 - I found a few hyperlinks that seem to link to other websites than described in the text:

- “With the possible exception of the schema used internally by Vivli and a few other specialist clinical data repositories” yet the link is to a CESSDA vocabulary collection https://vocabularies.cessda.eu/vocabulary/ModeOfCollection
- “is available as an enhanced DDI profile. CESSDA also offers tools for ensuring that the metadata is CDC valid” The link is https://cmv.cessda.eu/profiles/cdc/ddi-2.5/1.0.4/profile.xm where I believe an “l” is missing. https://cmv.cessda.eu/profiles/cdc/ddi-2.5/1.0.4/profile.xml does work however I believe a newer version of the CDC profile is available. If this is done explicitly as this profile was used for comparison it would be good to state this. I would find it even more useful if a link to an overview page is given rather than directly to the XML.
- “The CDC is part of the EOSC (European Open Science Cloud) marketplace and has recently developed a search API ( https://api.tech.cessda.eu/” although the text says something different, the hyperlink goes to https://bioportal.bioontology.org/ontologies/OCRE/
- When referring to the crosswalk, I would mention the DOI ( https://doi.org/10.5281/zenodo.8129621) instead of the Zenodo url ( https://zenodo.org/record/8129621).

- As a lover of PIDs, I would love to encourage Steve Canham, Katja Moilanen and Maria Panagiotopoulou to include an ORCID ID that can be linked to their authorship on this paper.

 - The text mentions that the article was created as part of the BY-COVID project and I was curious if the grant information is also included in the articles metadata.

Is the study design appropriate and does the work have academic merit?

Yes

Is the work clearly and accurately presented and does it cite the current literature?

Yes

If applicable, is the statistical analysis and its interpretation appropriate?

Not applicable

Are all the source data underlying the results available to ensure full reproducibility?

Yes

Are the conclusions drawn adequately supported by the results?

Partly

Are sufficient details of methods and analysis provided to allow replication by others?

Partly

Reviewer Expertise:

Social Sciences, Data Archiving Services

I confirm that I have read this submission and believe that I have an appropriate level of expertise to confirm that it is of an acceptable scientific standard, however I have significant reservations, as outlined above.

# Reviewer report: Reviewer response for version 1

**DOI:** 10.21956/openreseurope.17579.r35765

**Published:** 2023-11-13

**Authors:** Veerle Van den Eynden

**Affiliations:** 1 KU Leuven, Leuven, Flanders, Belgium

This paper provides a valuable comparison of metadata schemas and discovery metadata characteristics for two data catalogues to investigate the feasibility of cross-disciplinary exploration of data resources to facilitate cross-disciplinary research. The paper takes the case of two data catalogues, CESSDA for the social sciences & humanities, and ECRIN for the health sciences and clinical trials, to examine comparability and divergence, and to formulate recommendations for potential future cohesion.

 Whilst the paper presents clear explanations and evidence, there are suggestions for improvement:

1. I miss a description of the aims and objectives of this piece of work.
2. It would be good to say something about the role of EOSC to facilitate such discovery of resources across disciplines in the introduction;
3. It may be worth pointing out that whilst disciplinary metadata schemas may develop to meet discipline-specific needs, they often have historically developed starting from a common basis like DataCite and Dublin Core. This is worth mentioning.
4. For the literature search (first step in methods), indicate which databases / portals were searched and which search terms were used (for reproducibility of the research)
5. Provide explanations of abbreviations when first used, e.g. GDPR, API.
6. I'm not sure the statements on page 4-5 that study-originated resources may not be easily identified outside the context of the study and may lack PIDs and unique titles (whilst for study independent resources it is the opposite) are accurate. If you have evidence for this, then provide it, or reference it. The CESSDA DC has over 15000 datasets with 'study' in the title. Which datasets resulting from studies are not easy to find?
7. Organise the results in such way that it is clear what corresponds with steps 1, 2 etc. described in the methods. 
8. Is the statement on page 6 that it is currently difficult to discover similar research types & designs across domains correct? What about portals like Web of Science and Google Scholar for cross-domain discovery?
9. For DDI specify that the CDC profile is based on DDI Codebook.
10. Should URL links in the text be replaced by references, e.g. for DDI, the Zenodo link for the metadata mapping, etc.?
11. When describing the results of Table 3, the text refers to topics a to l. These letters are not used in the table. 
12. For suggestion 3 (source entities have different level of detail), what would / could be a solution?

Is the study design appropriate and does the work have academic merit?

Partly

Is the work clearly and accurately presented and does it cite the current literature?

Yes

If applicable, is the statistical analysis and its interpretation appropriate?

Not applicable

Are all the source data underlying the results available to ensure full reproducibility?

Yes

Are the conclusions drawn adequately supported by the results?

Yes

Are sufficient details of methods and analysis provided to allow replication by others?

Partly

Reviewer Expertise:

research data management; data sharing: open science; researcher practices: researcher attitudes & motic-vations

I confirm that I have read this submission and believe that I have an appropriate level of expertise to confirm that it is of an acceptable scientific standard, however I have significant reservations, as outlined above.

# Reviewer report: Reviewer response for version 1

**DOI:** 10.21956/openreseurope.17579.r35760

**Published:** 2023-11-13

**Authors:** Adrian Dusa

**Affiliations:** 1 University of Bucharest, Bucharest, Romania

This manuscript is a welcome and much awaited piece that attempts to promote the usage of secondary data through enhancing and stimulate the usage of metadata standard.

 It does so by making a highly interesting comparison between two of the most important research infrastructures in Europe: ECRIN and CESSDA.

 There are potentially unlimited possibilities of cross-domain research between the medical / clinical research and the social sciences, with a massive and deep impact over the whole European region.

 I very much welcome this type of publications, and hope this will be circulated as widely as possible.

 The manuscript is well written, using a clear and accessible language for any interested reader. I did not spot any obvious weakness in the text, and will therefore recommend it for publication.

 If anything, I would only have a couple of suggestions, if not for the current form of the paper then for (perhaps) future similar ones.

 First, sometimes the text seems to over employ the acronyms of the specific institutions, standards and procedures. They are of course presented earlier in the paper, but if the (especially) not specialised reader sometimes forgets what the acronym stands for, it is necessary to travel back to the original (first) mention of acronym. I would perhaps make an Annex with a list of all presented acronyms.

 Second and most important, the manuscript is obviously written by professional staff with proven and long standing expertise in these fields. The readers, however, are not always experts. They can be part of different scientific areas, or even regular people from the general public. The text should, therefore, take this into consideration and perhaps be made readable for a more wider audience. I had no problem whatsoever understanding the text, but on the other hand I do have expertise in this field.

 Third and perhaps more general, the context of this paper seems, yet again, addressed by professionals to professionals in the field. Science, however, has experienced an unprecedented opening, not only due to the open research but more because of involving the regular citizen, thus expanding far beyond the traditional research institution centred approach.

 The tools mentioned in this paper are so important that (I believe) deserve a much wider impact.

 Otherwise, the paper is well worthy of publication and I highly recommend it.

Is the study design appropriate and does the work have academic merit?

Yes

Is the work clearly and accurately presented and does it cite the current literature?

Yes

If applicable, is the statistical analysis and its interpretation appropriate?

Not applicable

Are all the source data underlying the results available to ensure full reproducibility?

Yes

Are the conclusions drawn adequately supported by the results?

Yes

Are sufficient details of methods and analysis provided to allow replication by others?

Yes

Reviewer Expertise:

Social research methodology, statistics, data archiving, metadata standards

I confirm that I have read this submission and believe that I have an appropriate level of expertise to confirm that it is of an acceptable scientific standard.
